# A longitudinal analysis of diet quality scores and the risk of incident depression in the SUN Project

**DOI:** 10.1186/s12916-015-0428-y

**Published:** 2015-09-17

**Authors:** Almudena Sánchez-Villegas, Patricia Henríquez-Sánchez, Miguel Ruiz-Canela, Francisca Lahortiga, Patricio Molero, Estefanía Toledo, Miguel A. Martínez-González

**Affiliations:** Nutrition Research Group, Research Institute of Biomedical and Health Sciences, University of Las Palmas de Gran Canaria, Las Palmas de Gran Canaria, Spain; Ciber de Fisiopatología de la Obesidad y Nutrición (CIBER OBN), Instituto de Salud Carlos III, Madrid, Spain; Department of Clinical Sciences, University of Las Palmas de Gran Canaria, P.O. Box 550, , CP. 35080 Las Palmas de Gran Canaria, Spain; Department of Preventive Medicine and Public Health, University of Navarra, C/ Irunlarrea, n° 1, CP. 31008 Pamplona, Spain; Department of Psychiatry and Medical Psychology, University Clinic of Navarra, Pamplona, Spain

## Abstract

**Background:**

Some studies have pointed out that several dietary patterns could be associated with a reduced risk of depression among adults. This association seems to be consistent across countries, cultures and populations. The objective of the study was to compare and to establish the type of relationship between three diet quality scores and depression in the SUN (Seguimiento Universidad de Navarra) Cohort study.

**Methods:**

We performed a dynamic cohort study based on Spanish university graduates free of depression at baseline. Dietary intake was repeatedly assessed at baseline and after 10 years of follow-up with a validated semi-quantitative food-frequency questionnaire. Three previously described diet quality scores: Mediterranean Diet Score (MDS), Pro-vegetarian Dietary Pattern (PDP) and Alternative Healthy Eating Index-2010 (AHEI-2010) were built. Participants were classified as having depression if they reported a new clinical diagnosis of depression by a physician or initiated the use of an antidepressant drug during follow-up. Time-dependent Cox regression models with cumulative averages of diet and restricted cubic splines were used to estimate hazard ratios of depression according to quintiles of adherence to the MDS, PDP and AHEI-2010.

**Results:**

One thousand and fifty one incident cases of depression were observed among 15,093 participants from the SUN Cohort after a median follow-up of 8.5 years. Inverse and significant associations were observed between the three diet quality scores and depression risk. The hazard ratios and 95 % confidence intervals for extreme quintiles (fifth versus first) of updated adherence to MDS, PDP and AHEI-2010 were 0.84 (0.69–1.02), 0.74 (0.61–0.89) and 0.60 (0.49–0.72), respectively. The dose–response analyses showed non-linear associations, suggesting that suboptimal adherence to these dietary patterns may partially be responsible for increased depression risk.

**Conclusions:**

Better adherence to the MDS, PDP and AHEI-2010 was associated with a reduced risk of depression among Spanish adults. However, our data suggested a threshold effect so that although the risk of depression was reduced when comparing moderate versus lower adherence, there was not much extra benefit for the comparison between moderate and high or very high adherence.

**Electronic supplementary material:**

The online version of this article (doi:10.1186/s12916-015-0428-y) contains supplementary material, which is available to authorized users.

## Background

Classically, one of the objectives of the nutritional epidemiology has been to analyse the role of diet in the prevention of some non-communicable diseases such as cardiovascular disease (CVD) or cancer and little attention has been paid to the effect on other diseases such as mental disorders [1]. However, an emerging field of research is currently being developed and several links between nutrition and mental health are being established. There are a number of scientifically rigorous studies making important contributions to the understanding of the role of nutrition in mental health [2]. Specifically, some studies have pointed out that several dietary patterns could be associated with a reduced prevalence [3, 4] and a reduced risk of depression among adults [4–10]. This association seems to be consistent across countries, cultures and populations according to several systematic reviews and meta-analyses [11–13]. Among these previously described dietary patterns are the Mediterranean Diet Score (MDS) [14], the Healthy Eating Index (HEI) [15, 16] and the Alternative Healthy Eating Index (AHEI) [17].

Although the scientific report of the 2015 Dietary Guidelines Advisory Committee has concluded that current evidence is limited, the protective dietary patterns associated with reduced risk of depression are those patterns emphasizing seafood, vegetables, fruits and nuts [18]. However, it is difficult to discern if differences in the intake of some micro- or macronutrients between these dietary patterns can make a difference in their association with a lower risk of depression.

Moreover, the type of association between adherence to these patterns and depression risk, and more specifically the shape of the dose–response curve and the potential existence of a non-linear threshold effect, has not yet been established.

For all of these reasons, the aims of this study were to compare the association of three diet quality scores with depression independently of their micro/macronutrients composition and to establish the type of relationship between these patterns and the risk of developing a depressive disorder within the SUN cohort study.

## Methods

### Study population

The SUN Project is a dynamic cohort study started on 21 December 1999. The aim of the SUN Project is to identify dietary and lifestyle determinants of hypertension, diabetes, obesity, coronary heart disease or depression, among other conditions. The participants of the study are former Spanish students of the University of Navarra, registered professionals from some Spanish provinces, and other university graduates. Baseline assessment and follow-up information is gathered through postal or web-based questionnaires collected every 2 years. Self-administered questionnaires include information regarding socio-demographic, lifestyle or dietary variables as well as the use of medication and the prevalence or incidence of different diseases during the follow-up. The overall retention in the cohort approaches 90 %. Further details about the methodology and characteristics of participants can be found in previously published reports [19, 20].

Up to June 2014, 22,045 participants had completed the baseline questionnaire of the SUN Project. Participants who were lost to follow-up, who had not completed at least one follow-up questionnaire, who were outside of predefined limits for energy intake (less than 800 kcal/d or more than 4000 kcal/d in men and less than 500 kcal/d or more than 3500 kcal/d in women), and those who were users of antidepressant medication or had reported a previous clinical diagnosis of depression at baseline were excluded from the analyses. After exclusions, 15,093 participants were included in this study.

The study was approved by the Institutional Review Board of the University of Navarra. Voluntary completion of the first questionnaire was considered to imply informed consent.

### Exposure assessment

Dietary intake was assessed at baseline and after 10 years of follow-up with a validated semi-quantitative food-frequency questionnaire [21, 22]. Nutrient intakes of 136 food items were calculated as frequency multiplied by nutrient composition of specified portion size for each food item, using an ad hoc computer program specifically developed for this aim. A trained dietician updated the nutrient database using the latest available information included in food composition tables for Spain [23].

The scoring criteria for the MDS, Pro-vegetarian Dietary Pattern (PDP) and AHEI-2010 are described in Table 1.

**Table 1:** The Mediterranean Diet Score, Pro-vegetarian Dietary Pattern and Alternative Healthy Eating Index-2010 scoring methods

Mediterranean diet score		Pro-vegetarian dietary pattern		Alternative healthy eating index-2010		
Component	Criteria	Component	Criteria	Component	Criteria for min score (0)	Criteria for max score (10)
Vegetables	1 point if consumption is above the sex-specific median	Vegetables	Energy-adjusted consumptions were ranked according to their sex-specific quintiles	Vegetables	0 serv/d	≥5 serv/d
Fruits and nuts		Fruits		Fruits	0 serv/d	≥4 serv/d
Cereals		Nuts		Whole-grain bread	0 g/d	420 g/d
Fish		Cereals		Sugar-sweetened beverages and fruit juice	≥1 drink/d	0 drinks/d
Legumes		Legumes				
Monounsaturated to saturated fatty acids ratio		Olive oil	Plant foods:	Nuts and legumes	0 serv/d	≥1 serv/d
		Potatoes	(Q1 = 1 point)	Red/processed meat	≥1.5 serv/d	0 serv/d
			(Q5 = 5 points)	Trans fatty acids	≥4 % E	≤0.5 % E
				Long-chain omega-3	0	≥250 mg/d
Meat and meat products	1 point if consumption is below the sex-specific median	Added animal fats	Energy-adjusted consumptions were ranked according to their sex-specific quintiles	Polyunsaturated fatty acids intake	≤2 % E	≥10 % E
		Eggs		Sodium	Highest decile	Lowest decile
Dairy products		Fish		Alcohol intake	≥3.5 drinks/d (men)	0.5–2.0 drinks/d (men)
		Dairy products				
(≥10 and ≤50 g/d) men	1 point if moderate alcohol intake	Meats and meat products	Animal foods:		≥2.5 drinks/d (women)	0.5–1.5 drinks/d (women)
(≥5 and ≤25 g/d) women			(Q1 = 5 points)			
			(Q5 = 1 point)			

#### Mediterranean Diet Score

Adherence to the Mediterranean diet was appraised according to the score proposed by Trichopoulou et al. [14]. This score includes nine components: vegetables, legumes, fruits and nuts, cereals, fish and seafood, meat and meat products, dairy products, moderate alcohol intake, and the ratio of monounsaturated fatty acids to saturated fatty acids. One point was assigned to persons whose consumption was at or above the sex-specific median of six components in agreement with the traditional Mediterranean diet (vegetables, fruits/nuts, legumes, fish/seafood, cereals, and monounsaturated to saturated lipid ratio). The participant received also 1 point if her or his intake was below the median for the two components not in line with the traditional Mediterranean diet (meat or meat products and dairy products). For ethanol, 1 point was assigned only for moderate amounts of intake (5–25 g/d for women or 10–50 g/d for men). Therefore, this score could range from the highest possible (9 points reflecting maximum adherence) to the minimum possible (0 points reflecting no adherence at all). Adherence to the MDS was categorized into five categories: low (score 0–2), low-moderate (score 3), moderate-high (score 4), high (score 5) and very high (6–9). This categorization was used to ensure an adequate distribution of the sample with enough participants in each category of adherence.

#### Pro-vegetarian Dietary Pattern

Because a pure vegetarian diet might not easily be embraced by many individuals, a moderate and intermediate approach to a vegetarian diet was proposed. This approach is the PDP, which was operationalized to quantify the habit of consuming preferentially plant-derived foods instead of animal-derived foods, but without the need to follow a strict vegetarian diet [24]. The PDP represents a more easily understood message than a pure vegetarian diet. To build the PDP, we adjusted the consumption of seven food groups from plant origin (fruit, vegetables, nuts, cereals, legumes, olive oil and potatoes) and five food groups from animal origin (added animal fats, eggs, fish, dairy products, and meats and meat products) for total energy intake by using the residual method separately for men and women, and created quintiles. The final score could range from 12 (lowest adherence) to 60 (highest adherence). Finally, this quantitative variable was categorized into quintiles [24].

#### Alternative Healthy Eating Index-2010

To build the AHEI-2010, 11 groups of foods or nutrients were considered: vegetables, fruits, whole-grain bread, sugar-sweetened beverages and fruit juice, nuts and legumes, red/processed meat, trans fatty acids, long-chain omega-3 fatty acids, polyunsaturated fatty acids, sodium, and alcohol intake [17]. Although the original AHEI-2010 includes the consumption of whole grains as one of its elements, its consumption is very scarce in the general Spanish population. In fact, almost the only source is a small amount of whole-grain bread that was considered as one of the elements in our score with nine categories of intake.

With the exception of whole-grain bread consumption, all AHEI-2010 components were scored from 0 (worst) to 10 (best). Thus, the total AHEI-2010 score could range from 0 (no adherence) to 109 (perfect adherence). This variable was categorized into quintiles.

### Outcome assessment

Incident cases of depression were defined as participants who were free of depression and not using antidepressant treatment at baseline who, in any of the follow-up questionnaires (Q_2-Q_14), positively responded to the following question “Have you ever been diagnosed with depression by a medical doctor?” or who reported the habitual use of antidepressant drugs.

A self-reported physician-provided diagnosis of depression demonstrated acceptable validity in a subsample of 104 participants of our cohort, using the Structured Clinical Interview for the Diagnostic and Statistical Manual of Mental Disorders fourth edition as the ‘gold standard’ applied by experienced psychiatrists blinded to the answers to the questionnaires [25]. There were 46 true positives of the 62 self-reported cases of depression. Thus, the percentage of confirmed depression was 74.2 %; (95 % confidence interval [CI] = 63.3–85.1). There were 34 true negatives of the 42 participants who did not report a depression diagnosis. Therefore, the percentage of confirmed non-depression was 81.1 % (95 % CI = 69.1–92.9).

### Other covariate assessment

Information on socio-demographic (e.g. sex, age, marital status, employment status) and lifestyle-related variables (e.g. smoking status, physical activity, use of vitamin supplements) were obtained from the baseline questionnaire (Q_0). Physical activity was assessed through a validated physical activity questionnaire with data about 17 activities [26]. Leisure-time activities were computed by assigning an activity metabolic equivalent score to each activity multiplied by the time spent in each activity, and summing up all activities. A participant was considered as a user of vitamin supplements if he or she reported at least the consumption of one of the following vitamin supplements: A, B_1_, B_2_, B_3_, B_6_, B_9_, B_12_, C, D or E.

Body mass index (BMI) was calculated as weight (in kilograms) divided by the square of height (in meters) using data collected at baseline and after 10 years of follow-up.

The prevalence and history of CVD, obesity, dyslipidaemia, hypertension (HTA) and type 2 diabetes mellitus (T2DM) was ascertained at baseline and updated until the end of follow-up or depression diagnosis was reported. CVD included myocardial infarction, stroke, atrial fibrillation, paroxysmal tachycardia, coronary artery bypass grafting or other revascularization procedures, heart failure, aortic aneurism, pulmonary embolism or peripheral venous thrombosis.

Energy intake also was calculated through the information collected from the semi-quantitative food-frequency questionnaire administered at baseline and after 10 years of follow-up.

### Statistical methods

For each participant we computed person-years of follow-up from the date of returning the baseline questionnaire to the date of depression diagnosis, the date of death or the date of returning the last follow-up questionnaire, whichever came first.

Cox proportional-hazards regression models were fitted to assess the relationship between the adherence to each of the different dietary patterns at baseline and the incidence of depression during follow-up. Hazard ratios (HRs) and their 95 % CIs were calculated considering the lowest quintile as the reference category. To control for potential confounding factors, the results were adjusted for sex, age (years, continuous), BMI (Kg/m^2^, continuous), smoking (non-smoker, ex-smoker, current smoker, missing), physical activity during leisure time (quintiles), use of vitamin supplements, total energy intake (kcal/d, continuous) and the presence of several diseases at baseline (CVD, T2DM, HTA and dyslipidaemia). Other confounding factors such as marital status and employment status were also explored but not included in the final models because their inclusion in the regression models did not substantially change the reported associations.

As a sensitivity analysis, we also took into account the year of recruitment and introduced it as a stratifying factor.

Tests of linear trend across increasing quintiles of adherence were conducted by assigning the medians to each quintile and treating it as a continuous variable.

To minimize any effect of variation in diet, we also calculated the average cumulative adherence to the different dietary patterns by using updated dietary scores with dietary data collected after 10 years of follow-up, and used time-dependent Cox models to compute the HRs. To increase accuracy, energy intake and BMI were also updated with the information obtained after 10 years of follow-up. The prevalence of diseases was updated using the information contained in any of the follow-up questionnaires.

To quantify the relationship between the adherence to different diet quality scores beyond the Mediterranean diet and depression, we fitted linear regression models with the PDP and the AHEI-2010 dietary indexes as the dependent variables and adherence to the MDS as the predictor. Model residuals (difference between observed scores and expected scores as predicted by MDS), which provide a measure of the adherence to the non-Mediterranean dietary patterns uncorrelated with the MDS, were categorized into quintiles and used as predictors of depression in new Cox models. To minimize the bias produced by measure units of each protective dietary pattern, z scores were used. Each z score was calculated as the score value minus the mean value for the score divided by standard deviation of the score.

Finally, the potential non-parametrical non-linear association between the cumulative average adherence to each of the dietary patterns and incident depression was calculated with restricted cubic splines [27]. Tests for non-linearity used the likelihood ratio test, comparing the model with only the linear term to the model with the linear and the cubic spline terms. The results were adjusted for the same potential confounding factors as the main Cox regression analysis.

All *P*-values were two-tailed and *P* < 0.05 was considered significant.

Statistical analysis was performed using STATA version 12.0 (StataCorp, College Station, TX, USA).

## Results

There were 1,051 incident cases of depression after a median follow-up of 8.5 years. Table 2 shows the distribution of baseline characteristics of participants according to extreme quintiles (first and fifth) of adherence to the three dietary patterns analysed in this study (MDS, PDP and AHEI-2010). Participants in the highest quintile of adherence to these dietary patterns were more likely to be married, were older, and showed a higher prevalence of CVD, T2DM or dyslipidaemia. Moreover, these participants were also more probably non-smokers and showed higher levels of physical activity during leisure time. Regarding energy intake, those participants belonging to the category of maximum adherence to the MDS showed the highest energy intake whereas those in the category of maximum adherence to the PDP and the AHEI-2010 reported lower energy intake.

**Table 2:** Characteristics (mean [SD] or percentage) of participants according to extreme quintiles of different diet quality scores

	Mediterranean		Pro-vegetarian		Alternative Healthy Eating Index-2010	
	Diet Score		Dietary Pattern			
	0–<3	6–9	Q1	Q5	Q1	Q5
	(n = 2,512)	(n = 3,427)	(n = 3,018)	(n = 3,019)	(n = 3,018)	(n = 3,018)
Age (years)	34.4	42.2	35.4	41.9	35.1	42.7
Male gender (%)	39.6	45.5	43.1	43.2	46.0	38.4
Marital status married (%)	44.6	58.8	47.0	57.7	46.1	57.8
Unemployed (%)	4.6	2.8	4.9	2.7	4.2	2.8
Smoking status (%)						
Ex-smoker	22.8	35.6	25.1	34.3	23.8	37.0
Current smoker	21.3	19.5	24.7	17.2	24.9	16.1
Use of vitamin supplements						
Supplements^a^ (%)	2.5	1.9	2.1	1.9	1.9	2.1
Special diet at baseline (%)	4.9	9.5	6.1	10.5	4.6	13.0
Post-menopause^b^ (%)	6.5	16.6	6.7	17.4	6.4	19.3
Prevalence of diseases (%)						
CVD	2.6	5.3	3.1	5.4	3.3	5.4
Cancer	3.0	4.6	3.0	4.1	2.5	4.7
T2DM	0.9	2.3	1.2	1.7	0.8	2.8
HTA	4.8	10.1	5.4	9.6	5.7	9.8
Dyslipidaemia	13.2	23.9	13.4	24.2	15.0	23.1
Obesity	4.1	4.7	4.7	4.1	5.0	3.8
Body mass index (kg/m^2^)	23.2	23.8	23.5	23.6	23.5	23.6
Energy intake (kcal/d)	2204	2512	2441	2361	2429	2254
Physical activity during leisure-time (METs-h/w)	18.4	25.3	20.3	23.7	19.1	25.0
Alcohol intake (drinks/d)	4.1	9.3	6.9	6.6	8.0	6.1
Mediterranean Diet Score	1.6	6.5	3.0	5.7	3.0	5.6
Pro-vegetarian Dietary Pattern	31.6	40.1	29.4	43.5	32.4	40.1
AHEI-2010	49.5	65.6	51.3	65.7	44.6	72.6

*Abbreviations*: *AHEI* Alternative Healthy Eating Index, *CVD* cardiovascular disease, *HTA* hypertension, *METs* metabolic equivalents, *Q1,Q5* Quintile1, Quintile5, *SD* standard deviation, *T2DM* type 2 diabetes mellitus

^a^Use of at least one of the following vitamin supplements: A, B_1_, B_2_, B_3_, B_6_, folic acid, B12, C, D or E

^b^8,847 women were included

The association between adherence to the MDS, PDP or AHEI-2010 and the risk of depression is shown in Table 3. Both analyses, those regarding baseline adherence and updated adherence after 10 years of follow-up, are shown in the table. A moderate adherence to the MDS at baseline was already associated with an important reduction in the risk of developing depression during the follow-up as compared to the minimum adherence. In fact, those participants in the second to fifth quintile of adherence showed relative risk reductions of 25–30 %. When changes in the adherence were taken into account (repeated measurements analysis), the relationship was attenuated although the dose–response relationship remained significant. The magnitude of the association was similar for the PDP; comparing participants in the highest quintile of adherence to the PDP with those in the lowest quintile, the multivariable HR was 0.78 (95 % CI = 0.64–0.93) in the analysis using baseline exposure and 0.74 (0.61–0.89) in the analysis with updated repeated measurements of the dietary pattern, both with significant linear trend tests. Finally, an inverse and significant association was observed for the adherence to the AHEI-10 and depression risk. In the analysis using repeated measurements, the HR and 95 % CI for successive quintiles of updated adherence to the AHEI-10 were 1 (ref.), 0.68 (0.57–0.82), 0.75 (0.63–0.90), 0.55 (0.46–0.67) and 0.60 (0.49–0.72), with a significant dose–response relationship (*P* for trend < 0.001).

**Table 3:** Risk of depression (HR and 95 % CI)^a^ according to the adherence to quintiles of different diet quality scores

Mediterranean Diet Score	0–<3	3–<4	4–<5	5–<6	6–9	*P* for trend
Cases	221	191	212	200	227	
Person-years	22,560	25,206	27,338	25,812	30,330	
Crude rates/10^3^ ^b^	9.8 (8.6–11.2)	7.6 (6.5–8.7)	7.8 (6.7–8.9)	7.7. (6.7–8.9)	7.5 (6.5–8.5)	
Baseline	1 (ref.)	0.74 (0.61–0.90)	0.76 (0.63–0.92)	0.74 (0.61–0.90)	0.70 (0.58–0.85)	0.002
Repeated measures^c^	1 (ref.)	0.79 (0.65–0.96)	0.76 (0.62–0.92)	0.80 (0.66–0.97)	0.84 (0.69–1.02)	0.001
Pro-vegetarian Dietary Pattern	Q1	Q2	Q3	Q4	Q5	
Median	30	34	36	39	43	
Cases	319	224	136	183	189	
Person-years	34,090	27,862	20,032	24,584	24,677	
Crude rates/10^3 b^	9.4 (8.4–10.4)	8.0 (7.0–9.2)	6.8 (5.7–8.0)	7.4 (6.4–8.6)	7.7 (6.6–8.8)	
Baseline	1 (ref.)	0.83 (0.70–0.99)	0.70 (0.57–0.86)	0.76 (0.63–0.91)	0.78 (0.64–0.93)	0.003
Repeated measures^c^	1 (ref.)	0.88 (0.74–1.04)	0.52 (0.43–0.64)	0.68 (0.57–0.82)	0.74 (0.61–0.89)	<0.001
Alternative Healthy Eating Index-2010	Q1	Q2	Q3	Q4	Q5	
Median	46	52	58	64	71	
Cases	282	197	229	160	183	
Person-years	30,175	24,041	30,488	21,979	24,564	
Crude rates/10^3 b^	9.3 (8.3–10.5)	8.2 (7.1–9.4)	7.5 (6.6–8.5)	7.3 (6.2–8.5)	7.4 (6.4–8.6)	
Baseline	1 (ref.)	0.84 (0.70–1.01)	0.77 (0.64–0.90)	0.73 (0.60–0.88)	0.72 (0.59–0.88)	<0.001
Repeated measures^c^	1 (ref.)	0.68 (0.57–0.82)	0.75 (0.63–0.90)	0.55 (0.46–0.67)	0.60 (0.49–0.72)	<0.001

*Abbreviations*: *CI* confidence interval, *HR* hazard ratio, *Q1-Q5* Quintile1-Quintile5

^a^Adjusted for age, sex, body mass index, smoking, physical activity during leisure time, use of vitamin supplement, total energy intake and presence of several diseases at baseline (cardiovascular disease, type 2 diabetes, hypertension and dyslipidaemia). For Pro-vegetarian Dietary Pattern, additionally adjusted for alcohol intake

^b^Crude rates and 95 % confidence intervals

^c^Repeated measures. Cumulative average for dietary patterns (at baseline and after 10 years of follow-up). Energy intake, body mass index and prevalence of diseases were also updated. For Pro-vegetarian Dietary Pattern, alcohol intake was also updated

Table 4 shows the association between z-PDP and z-AHEI-2010 residuals on the z-MDS and depression risk. The variables used as exposures in these analyses were residuals of a linear regression model where the dependent variable was PDP and the independent variable was MDS, and residuals of a linear regression model where the dependent variable was AHEI-2010 and the independent variable was MDS. Therefore, both exposures (residuals from these regressions) captured the variability in the respective scores (PDP and AHEI-2010) that were not explained by adherence to the Mediterranean diet. The most important reduction in the risk of depression associated with adherence to the AHEI-10 could be explained by the correlation of this pattern with the MDS (HR for third quintile versus first quintile = 0.69; 95 % CI = 0.57–0.83). The median z-AHEI 2010 residual for the third quintile was 0.01, which represents the AHEI-2010 adherence that could be explained by MDS (if 0 both patterns would be totally correlated). So, beyond the MDS, the magnitude of the effect of AHEI-10 on depression seemed to be lower, because whenever the correlation between dietary patterns was lower, the risk reduction was also of lower magnitude. This effect was less apparent for the PDP.

**Table 4:** Risk of depression (HR and 95 % CI)^a^ for the quintiles of the residuals of z-Pro-vegetarian Dietary Pattern and z-Alternative Healthy Eating Index-2010 on the z-Mediterranean Diet Score

z-Pro-vegetarian Dietary Pattern	Q1	Q2	Q3	Q4	Q5
Median	−1.01	−0.41	0.02	0.46	1.08
Cases	251	193	224	188	195
Person-years	27,451	26,224	27,173	25,535	24,863
Baseline residual	1 (ref.)	0.79 (0.66–0.96)	0.89 (0.74–1.06)	0.80 (0.66–0.97)	0.84 (0.69–1.02)
Repeated measures^b^	1 (ref.)	0.82 (0.68–0.99)	0.88 (0.73–1.06)	0.79 (0.65–0.95)	0.82 (0.68–0.99)
z-Alternative Healthy Eating Index-2010	Q1	Q2	Q3	Q4	Q5
Median	−1.07	−0.43	0.01	0.43	1.07
Cases	252	217	190	180	212
Person-years	27,046	26,577	26,836	25,487	25,300
Baseline residual	1 (ref.)	0.86 (0.71–1.03)	0.74 (0.61–0.89)	0.72 (0.59–0.87)	0.85 (0.70–1.03)
Repeated measures^b^	1 (ref.)	0.82 (0.68–0.99)	0.69 (0.57–0.83)	0.67 (0.55–0.81)	0.77 (0.64–0.93)

*Abbreviations*: *CI* confidence interval, *HR* hazard ratio, *Q1-Q5* Quintile1-Quintile5

^a^Adjusted for age, sex, body mass index, smoking, physical activity during leisure time, use of vitamin supplement, z scores for Mediterranean Diet Score, total energy intake and presence of several diseases at baseline (cardiovascular disease, type 2 diabetes, hypertension and dyslipidaemia)

^b^Repeated measures. Cumulative average for dietary patterns (at baseline and after 10 years of follow-up). Energy intake, body mass index and prevalence of diseases were also updated. For Pro-vegetarian Dietary Pattern, alcohol intake was also updated

**Fig. 1:**
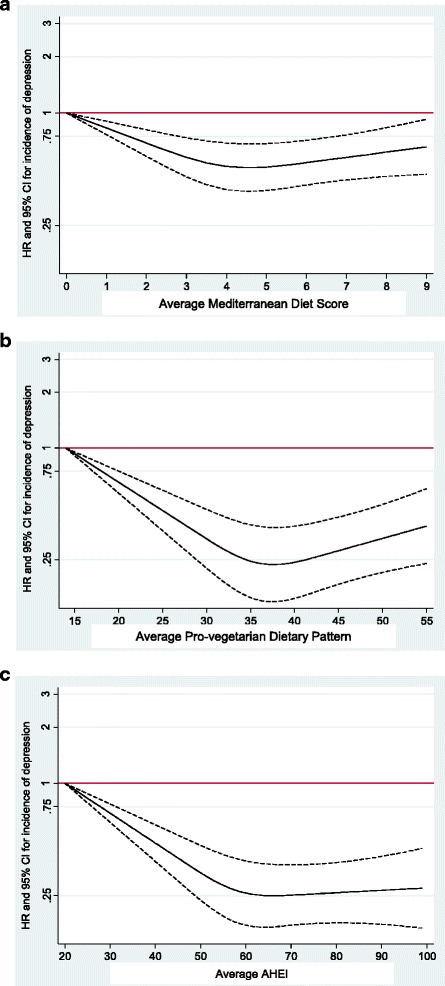
Spline regression models. **a** Spline regression model of the relative risk of depression according to adherence to the Mediterranean Diet Score (the dotted lines represent 95 % confidence intervals). **b** Spline regression model of the relative risk of depression according to adherence to the Pro-vegetarian Dietary Pattern (the dotted lines represent 95 % confidence intervals). **c** Spline regression model of the relative risk of depression according to adherence to the Alternative Healthy Eating Index-2010 (the dotted lines represent 95 % confidence intervals)

To account for non-linear associations, we used restricted cubic spline analysis. We found a suggestion of L-shaped associations (Fig. 1), indicating that moving from low to moderate adherence to these diet quality scores should be responsible for a reduction in the risk of depression. An apparent threshold effect was found, so that no extra benefit beyond moderate adherence was observed in potential comparisons of high or very high adherence versus moderate adherence.

## Discussion

The results of this study suggest that moderate or high adherence to diet quality scores such as the MDS, the PDP or the AHEI-2010 could be effective for the reduction of depression risk. This is, to our knowledge, the first time that several dietary patterns reflecting overall diet quality have been compared in the same cohort in relation to depression risk, and non-linear associations explored.

We considered these three diet quality scores for multiple reasons. First, several studies have prospectively analysed the role of the Mediterranean diet in total mortality [28, 29] and in several diseases such as CVD [30–32], T2DM [33, 34], HTA [35, 36] and even depression [4, 8–10]. Regarding the effect of the PDP, the PREDIMED study showed an inverse association between PDP and total mortality [24]. With respect to the AHEI-2010, an inverse association was found with total, cardiovascular or cancer mortality [37] and with several diseases, such as T2DM [38], in a longitudinal analysis within the Multiethnic Cohort study.

Second, to our knowledge, there are no prospective studies that have analysed the contribution of the PDP or the AHEI-2010 to the potential prevention of depression. Only a few studies have analysed the association between the AHEI-2005, the HEI or empirically-derived food patterns and depression. Pagoto et al*.* found that higher scores in depressive symptoms collected through the Center for Epidemiologic Studies Depression Scale were associated with lower diet quality (measured through the AHEI-2005) in a cross-sectional study among Latinos at risk of T2DM [39]. Regarding prospective studies, only a study by Akbaraly et al*.*, also based on the AHEI-2005, found a lower risk of depression associated with a higher adherence to the pattern, and only among women [5]. Those women who maintained or even improved their adherence to this dietary pattern along 10 years showed a significant reduction (around 65 %) in the risk of developing depressive symptoms as compared to those with low adherence. Other hypothesis-oriented patterns such as the HEI have also been associated with lower depressive symptoms in cross-sectional studies [40, 41], whereas the results related with a posteriori patterns (empirically derived food patterns) have been inconsistent in both cross-sectional [7, 42] and longitudinal studies [7, 43, 44].

A third reason to analyse the AHEI-2010 is that we consider the AHEI-2010 to update previous indexes, such as the AHEI-2005 or the HEI, and to add new relevant information. In fact, the AHEI-2005 and AHEI-2010 differ substantially in the items used in their scoring. For example, multivitamin use is not included in the AHEI-2010 whereas sodium or omega-3 fatty acid intake and sugar-sweetened beverage consumption are not considered in the AHEI-2005 but are included in the 2010 version.

The protective role of several dietary patterns against chronic diseases including depression can be explained through their nutritional properties. The three dietary patterns analysed in this study negatively weighted food items such as meat, meat products and sweets (sources of animal fats: saturated and trans fatty acids). In contrast, several food items, such as nuts (source of omega-3 fatty acids), fruits, legumes and vegetables (source of vitamins and minerals), were positively weighted.

In this context, although several prospective studies have recently associated the intake of some nutrients and food items such as trans fatty acids, sweets and bakery or fast food with an increased risk of developing depression [5, 6, 45, 46], other longitudinal studies that have analysed the role of the omega-3 fatty acids or the omega-3/omega-6 ratio [46–48] and some micronutrients such as B-vitamins and folate [49–52], vitamin E [53] or minerals such as magnesium [54] or zinc [55, 56] in depression prevention have reported inconsistent results. For example, although an inverse association was observed between omega-3 fatty acids intake and depression in a preliminary analysis within the SUN Project [48], no association was revealed in subsequent analysis with a longer follow-up period and a higher sample size [46]. While Tolmunen et al*.* found that low dietary folate intake could be considered a risk factor for depression [52], recent longitudinal studies failed to find a significant association between folate or other B vitamin supplementation and depression incidence [49, 51]. Regarding magnesium intake, a cross-sectional study published by Jacka in 2009 reported an inverse association between magnesium intake and depressive symptoms [57] although reverse causality could explain the reported result. But, no statistically significant association between magnesium intake and depression risk was found in the SUN cohort study in a prospective analysis [54]. Prospective cohort studies that have evaluated the role of zinc intake in depression risk also showed conflicting results. Whereas low dietary zinc intake was not longitudinally associated to depression in the Kuopio Ischemic Heart Disease Risk Factor Study [55], an inverse and significant association was observed in two large longitudinal studies of middle-age and older Australians [56].

One possible explanation of the different magnitude of effect found for the three dietary patterns (MDS, PDP and AHEI-2010) on depression risk is their distinct nutritional composition, differing in vitamin, mineral and macronutrient content. For example, as we have already mentioned above, all of them are important sources of vitamins and minerals (vegetables, fruits, nuts or legumes). However, the MDS is rich in monounsaturated fatty acids and fish (omega-3 fatty acids) and the AHEI-2010 in nutrients such as polyunsaturated fatty acids (both omega-3 and omega-6 fatty acids). Moreover, the consumption of fish (the most important source of long-chain omega-3 fatty acids) is negatively scored in the PDP. Finally, the AHEI-2010 takes into account sodium intake or the consumption of sugar- sweetened beverages.

One factor seems to be common to all the findings we observed. Moderate adherence (but not always the highest level of adherence) to diet quality scores showed the strongest inverse association with depression. It could be speculated that some psychological elements of neurotic or obsessive traits present in some participants classified in the highest category of dietary adherence may contribute to the observed plateau reached at moderate adherence. An alternative explanation for this plateau is that we observed suboptimal intakes for some micronutrients: vitamin E, folic acid and magnesium (below the recommended daily allowance) in participants within the first quintile of adherence to the three dietary patterns (Additional file 1: Table S1). Therefore, a threshold effect may exist, and once the threshold is achieved the risk reduction with subsequent improved adherence plateaued. This explanation is also compatible with our observed results. In fact, an accrual of studies based on patients with symptoms of depression has found suboptimal intakes of these micronutrients among these patients. Moreover, not only intake, but also low serum levels of folate, zinc or magnesium have also been associated with depressive symptoms in other cross-sectional studies [58–61].

Finally, we have to highlight that, taking into account the observed correlation between the Mediterranean diet and the PDP, the PDP showed additional and relevant information on the association between diet quality scores and depression risk, because the residuals of a regression of PDP on the Mediterranean diet were still significantly associated with depression risk. This was not the case for the AHEI-2010. We observed a lower magnitude for the reductions in the risk of depression associated with the AHEI-2010 once we removed the variability in this score already explained by the Mediterranean diet. This latter finding suggests that common nutrients and food items present in both patterns (AHEI-2010 and the Mediterranean diet) could be responsible for the observed reduced risk in depression associated with a good adherence to the AHEI-2010.

Some strengths of our study are its prospective design with a high retention rate; the inclusion of a large number of participants; the existence of published validation studies regarding some of the most important co-variables, including the exposure and the outcome; and the use of repeated measurements of the dietary patterns. Some limitations of our study should also be acknowledged. A self-reported dietary intake and a self-reported clinical diagnosis of depression were used to define the main variables of the analysis. Although both assessments were validated in subsamples of participants of the cohort [21, 25], some degree of misclassification might still exist. However, this misclassification is more likely to be non-differential, and therefore would bias the results towards the null. Another possible caveat might be inherent residual confounding because of the possibility that some confounding variables were measured imperfectly or with some error or that some unknown or unmeasured confounders related to lifestyle might also have biased our reported results. Finally, our participants are not representative of the general Spanish population. We restricted our cohort to highly educated participants to obtain a better quality of self-reported information, to improve the retention rate, and to minimize confounding by educational level, and therefore by socio-economic status.

## Conclusions

Better adherence to the three diet quality scores was associated with a reduced risk of depression among Spanish adults. However, it seems that after eliminating the possible influence of the MDS, the AHEI-2010 shows a considerably weaker inverse association with depression risk. Interestingly, our observed dose–response relationships did not suggest a linear pattern. Instead, a threshold effect was apparent, with substantial risk reductions with moderate adherence compared to low adherence to quality dietary patterns, but almost no further extra benefit with subsequent improvements from moderate to maximum adherence. This dose–response pattern is compatible with the hypothesis that suboptimal intake of some nutrients (mainly located in low adherence levels) may represent a risk factor for future depression. More studies analysing the role of the adequacy of nutrient intake to meet neurophysiological requirements and the role of suboptimal levels of micronutrients in depression risk are needed to explore this possible dose–response pattern. Also, further large prospective studies and trials to confirm this hypothesis are needed to provide effective population strategies for using dietary patterns in the prevention of depression.

## Additional file

Additional file 1:
**Micronutrient intake within the lowest quintile of adherence to different diet quality scores.** Suboptimal intakes. (DOC 34 kb)

## Abbreviations

BMI: body mass index
CI: confidence interval
CVD: cardiovascular disease
HR: hazard ratio
HTA: hypertension
MDS: Mediterranean diet score
MET: hetabolic equivalent
PDP: Pro-vegetarian Dietary Pattern, AHEI, Alternative Healthy Eating Index
RDA: recommended daily allowance
SUN: Seguimiento Universidad de Navarra
T2DM: Type 2 diabetes mellitus
