# Supplementary material for: A longitudinal analysis of diet quality scores and the risk of incident depression in the SUN Project
Source: BMC Med. 2015 Sep 17;13:197. doi: 10.1186/s12916-015-0428-y (PMC4573281; doi:10.1186/s12916-015-0428-y)
Supplement: Additional file 1: — Micronutrient intake within the lowest quintile of adherence to different diet quality scores. Suboptimal intakes. (DOC 34 kb) [file 12916_2015_428_MOESM1_ESM.doc]

**Additional file 1: Table S1.** Micronutrient intake within the lowest quintile of adherence to different diet quality scores. Suboptimal intakes

|  | Men | | | | Women | | | |
| --- | --- | --- | --- | --- | --- | --- | --- | --- |
| Average intake | Q1 MDS | Q1 PDP | Q1 AHEI | RDA^a^ | Q1 MDS | Q1 PDP | Q1 AHEI | RDA^a^ |
| Vit A (µg/d) | 1107.79 | 1381.36 | 1269.12 | 900 | 1401.04 | 1749.27 | 1474.31 | 700 |
| Vit C (mg/d) | 165.07 | 202.88 | 194.39 | 90 | 196.80 | 239.56 | 215.31 | 75 |
| Vit E (mg/d) | 5.78 | 6.69 | 6.05 | 15 | 6.18 | 6.86 | 6.14 | 15 |
| Vit B12 (µg /d) | 8.90 | 11.57 | 9.72 | 2.4 | 8.35 | 10.74 | 8.73 | 2.4 |
| Folate (µg day) | 273.07 | 331.77 | 313.80 | 400 | 306.65 | 366.47 | 331.60 | 400 |
| Vit B6 (mg/d) | 2.12 | 2.59 | 2.37 | 1.3 | 2.21 | 2.65 | 2.34 | 1.3 |
| Magnesium (mg/d) | 335.74 | 391.40 | 374.90 | 420 | 345.44 | 390.49 | 360.51 | 320 |
| Zinc (mg/d) | 14.52 | 17.23 | 15.88 | 11 | 15.87 | 18.39 | 16.24 | 8 |

Abbreviations: MDS Mediterranean Diet Score, PDP Pro-Vegetarian Dietary Pattern, AHEI Alternative Healthy Eating Index, Q1 Quintile 1, *RDA* recommended dietary allowances

Bold text and numbers refer to micronutrients with suboptimal intakes
